# Malignant Peripheral Nerve Sheath Tumor Arising After Superficial Neurofibroma Excision: A Rare Sporadic Case in a Patient Without Neurofibromatosis Type 1

**DOI:** 10.7759/cureus.95846

**Published:** 2025-10-31

**Authors:** Maximillion W Hayama, Lindsey Marian, Kali Morrissette, Jennifer Crimmins, Michelle Pavlis

**Affiliations:** 1 Dermatology, Duke University School of Medicine, Durham, USA; 2 Pathology, Duke University Medical Center, Durham, USA; 3 Dermatology, Duke University Medical Center, Durham, USA

**Keywords:** cutaneous neurofibroma, malignant peripheral nerve sheath tumour, mpnst, neurofibroma, neurofibromatosis 1

## Abstract

Malignant peripheral nerve sheath tumors (MPNSTs) are rare sarcomas that often arise in the setting of neurofibromatosis type 1 (NF1) or prior radiation exposure. Sporadic MPNSTs are significantly less common and rarely originate from superficial cutaneous neurofibromas. We present a unique case of MPNST arising at the site of a previously excised superficial neurofibroma in a patient without NF1. This case highlights the diagnostic complexity of MPNSTs arising in superficial cutaneous neurofibromas, requiring timely recognition given the tumor’s aggressive behavior and poor prognosis. While degenerative changes within neurofibromas are typically benign, they may obscure early malignant transformation, complicating diagnosis. These findings underscore the need for continued surveillance of recurrent neurofibromas and further research into MPNST pathogenesis in non-NF1 populations.

## Introduction

Malignant peripheral nerve sheath tumors (MPNSTs) are rare and aggressive soft tissue sarcomas that typically arise from peripheral nerves or preexisting neurofibromas, most commonly in the context of neurofibromatosis type 1 (NF1). Approximately 10% of NF1 patients develop an MPNST, and over half of all MPNSTs are associated with NF1 mutations [1]. Sporadic MPNSTs in patients without NF1 or prior radiation exposure are significantly less common and more frequently arise from deep-seated plexiform neurofibromas located in the torso or proximal extremities [2]. Even more rare are MPNSTs arising from superficial cutaneous neurofibromas, particularly in individuals without NF1. Prediction of malignant transformation, typically arising from plexiform neurofibromas, is both clinically and histologically challenging due to the tumors’ often insidious evolution [3, 4]. Given these diagnostic challenges, we present a rare case of low-grade MPNST arising at the site of a previously excised superficial neurofibroma in a patient without NF1.

## Case presentation

**Figure 1:**
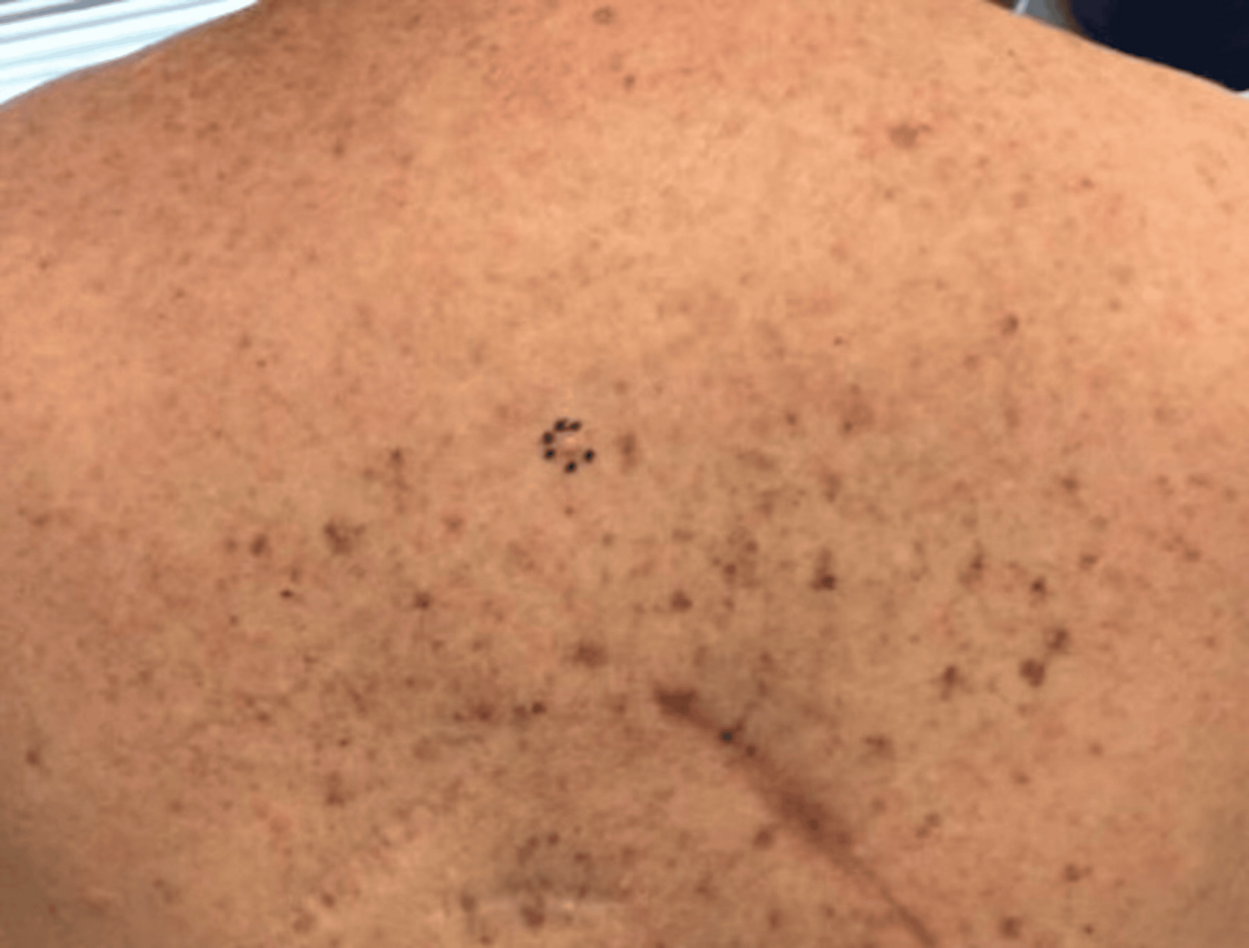
Clinical images An 8mm pink, dome-shaped papule on the mid-upper back (circumscribed by a marking pen).

**Figure 2:**
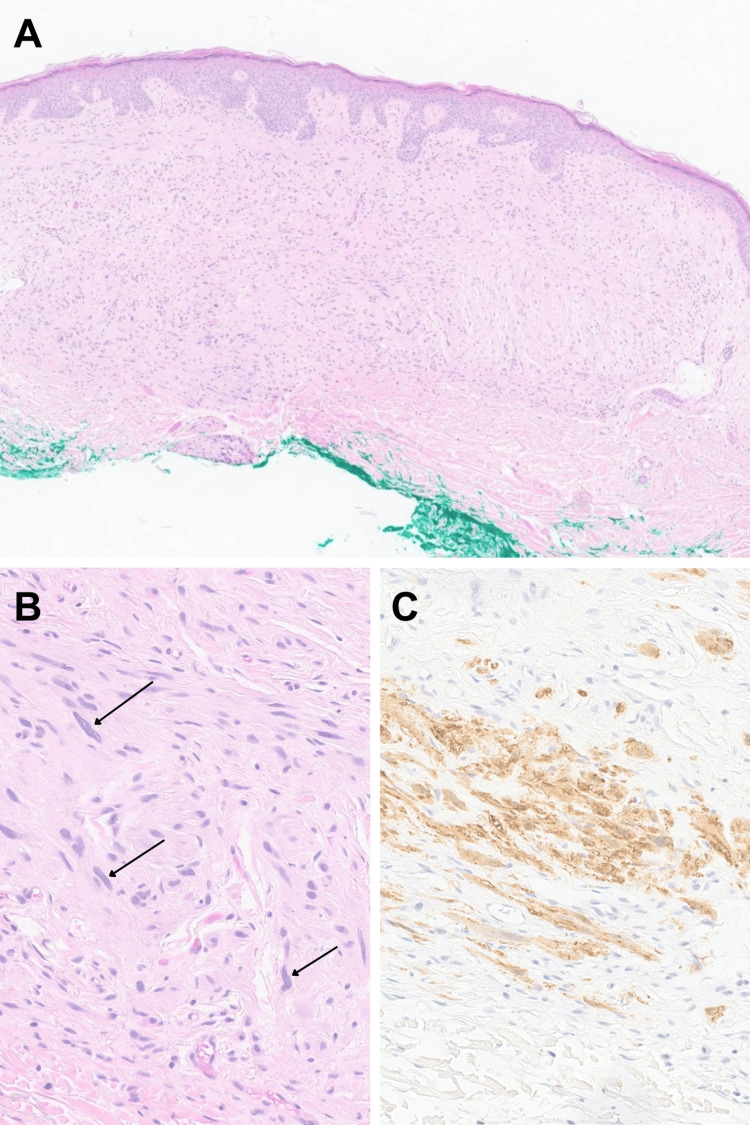
Histopathology of initial neurofibroma (A) Low power examination (H&E, 4x) demonstrates a well-delineated, relatively hypocellular dermal-based spindle cell proliferation in a background of fibrotic stroma. (B) Higher power examination (H&E, 20x) demonstrates most of the cells are bland-appearing, spindle-shaped with tapered nuclei. There are occasional moderately enlarged cells with smudgy chromatin (arrow). Mitotic figures were inconspicuous. (C) Immunohistochemistry (S100, 20x) is positive (expressed in cells of neural crest origin) in the lesion (brown).

A woman in her 60s with a history of multiple primary cutaneous melanomas, including in situ and stage 1a lesions treated with wide local excisions, presented to dermatology for evaluation of a lesion on her mid-upper back that had been intermittently irritated for several months. On examination, the lesion appeared as an 8mm pink dome-shaped papule (Figure 1). Given its persistent irritation, a shave biopsy was performed. Histopathology revealed a dermal-based spindle cell proliferation with scattered low-grade cytologic atypia, attributed to ancient-type changes, and no significant mitotic activity: features consistent with a neurofibroma (Figures 2A, 2B). The lesion was present at the deep tissue edge. Immunohistochemical staining was diffusely positive for S100 and SOX10, supporting the diagnosis (Figure 2C). Given these benign features, a re-excision was not performed, and routine monitoring was advised. 

**Figure 3:**
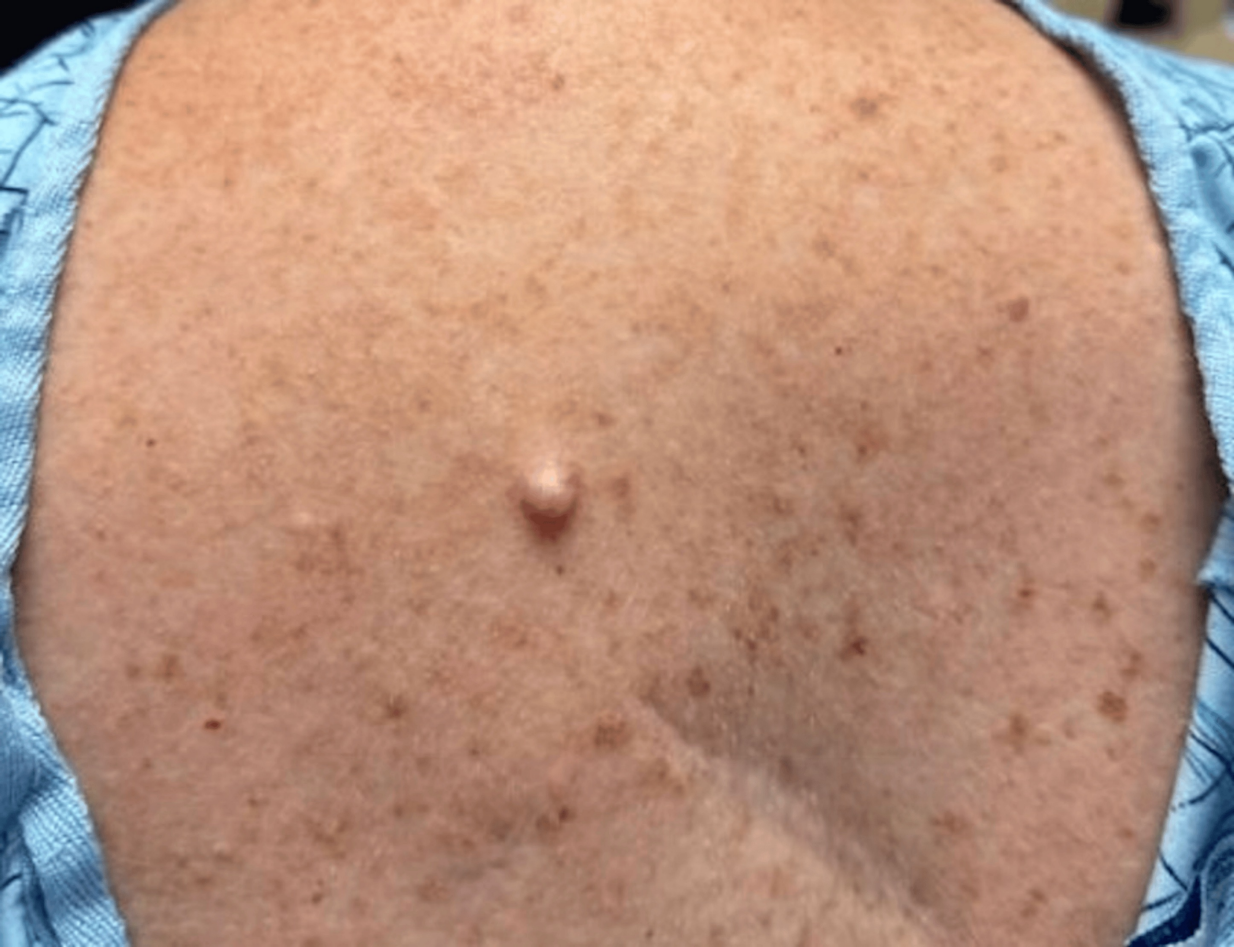
Clinical image 3 years later (B) A larger 1.3cm pink, dome-shaped papule on the mid-upper back approximately 3 years later.

**Figure 4:**
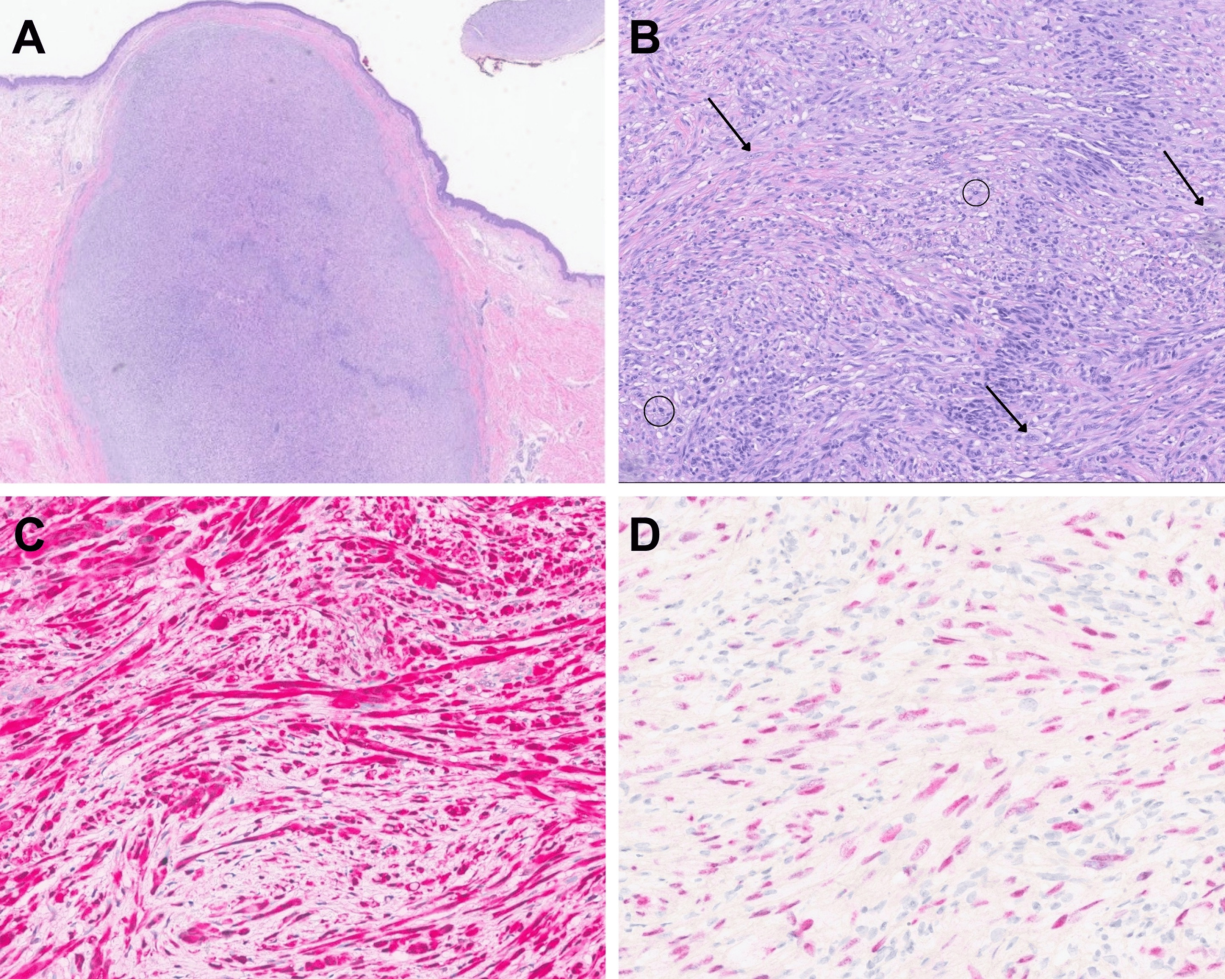
Histopathology of recurrent superficial MPNST (A) Low power examination (H&E, 2x) shows a dermal-based, well-circumscribed proliferation with increased cellularity. (B) Higher power examination (H&E, 10x) shows a haphazard to fascicular spindle cell proliferation with increased cellularity throughout, nuclear pleomorphism (arrows) and mitotic activity (circles). (C) Immunohistochemistry (S100, 20x) showed relatively diffuse expression in the lesion (red). (D) Immunohistochemistry (SOX10, 20x) is positive (expressed in cells of neural crest origin) in the lesion (patchy expression, red).

Three years later, she returned with a recurrent lesion in the same anatomical location, reporting new-onset tenderness and gradual enlargement over several months. Examination revealed a larger, 1.3cm, pink, firm, dome-shaped papule at the same site (Figure 3). She initially opted for observation but returned six months later with worsening discomfort and requested removal. A repeat excisional biopsy revealed a dermal spindle cell neoplasm with a sheath-like growth pattern at low power and increased nuclear atypia and mitotic activity compared to the previous specimen, without evidence of overlying junctional melanocytic proliferation (Figures 4A, 4B). Immunohistochemistry demonstrated S100 and SOX10 positivity (Figures 4C, 4D).

Based on the histologic and immunophenotypic findings in the setting of a previous neurofibroma in this anatomic site, the lesion was most consistent with a low-grade malignant peripheral nerve sheath tumor. A CT scan of her chest was negative for metastatic disease. The patient underwent local excision, with two-centimeter clear, radial margins. Given the superficial, low-grade characteristics of the tumor with no evidence of measurable disease on imaging, there were no indications to pursue further treatment with sentinel lymph node biopsy or adjuvant radiotherapy. Germline testing using a comprehensive CancerNext Panel (including NF1, CDKN2A, TP53, BRCA1/2, and MITF) revealed no pathogenic variants or variants of uncertain significance. She remains under close follow-up with both dermatology and surgical oncology at six-month intervals, with routine skin exams and CT scans for surveillance and evaluation of possible recurrence or metastasis. 

## Discussion

MPNSTs are rare soft tissue sarcomas, estimated to occur in approximately 0.0001% of the general population (1.46 per 1,000,000 people per year) [5]. Although a strong association exists between MPNSTs and NF1, approximately 45% of cases arise sporadically due to somatic mutations such as inactivation of the NF1 gene or loss of tumor suppressor genes like CDKN2A, TP53, and PTEN [1, 6]. This underscores the potential for these tumors to develop from pre-existing neurofibromas or nerve sheath elements even in patients without a germline NF1 mutation. In our patient, saliva germline testing did not reveal mutations in NF1 or other cancer-predisposing genes; however, routine clinical testing possesses several limitations and may miss structural variants or mutations in genes not included on targeted panels, limiting its ability to fully rule out an underlying genetic driver [7]. 

While transformation of plexiform neurofibromas into MPNST is well documented in NF1 patients, malignant change in cutaneous neurofibromas among individuals without NF1 is rare and poorly understood [7]. One study reported four cases of superficial MPNST in non-NF1 patients [8], while another found only one such case among 13 patients with superficial MPNST [9]. These lesions typically demonstrated spindle cell morphology with increased mitotic activity and variable S-100 expression, features closely resembling those observed in our patient [8, 9]. Furthermore, instances of MPNSTs arising from previously excised neurofibromas are exceedingly rare, especially in patients without NF1. One case series documented three such instances, each occurring after numerous excisions of locally recurrent, superficial, extensive neurofibromas [10]. In contrast, our case describes MPNST development following the initial excision of a superficial, focal neurofibroma.

Standard treatment for MPNST involves wide surgical excision with two-centimeter margins, and adjuvant radiotherapy may be considered for high-grade tumors or positive margins. Despite treatment, MPNSTs carry a high recurrence rate (43%) and a 10-year survival rate of approximately 50% [11, 12]. Although superficial MPNSTs may be detected earlier due to their anatomic accessibility, their outcomes remain comparable to deeper tumors in terms of metastasis and recurrence [8]. Furthermore, high-grade MPNSTs, which are differentiated from low-grade counterparts by increased hypercellularity, mitotic figures (often >10 per 10 high-power fields), and geographic necrosis, have been associated with significantly worse overall survival compared to low-grade MPNSTs [13]. 

A superficial MPNST arising from a previously excised cutaneous neurofibroma in an NF1-negative patient is rare and diagnostically challenging. In the absence of established clinical or histologic predictors, malignant transformation may go unrecognized until progression is more advanced. One complicating factor includes the presence of degenerative (also referred to as "ancient") changes within neurofibromas: features such as nuclear hyperchromasia, pleomorphism, and stromal hyalinization [4]. While typically regarded as benign, these changes can obscure or mimic early malignant progression, particularly in longstanding or recurrent lesions. Although both ancient-type changes in benign neurofibromas and early malignant transformation in MPNSTs may demonstrate nuclear atypia, MPNSTs are further delineated by increased cellularity, mitotic activity, and architectural disruption [4]. These challenges highlight the importance of long-term surveillance for recurrent or symptomatic neurofibromas, even in patients without known risk factors. Continued research into the pathogenesis and early detection of MPNSTs in non-NF1 populations is essential to improve outcomes and guide clinical decision-making.

## Conclusions

This case highlights the rare occurrence of a superficial MPNST arising at the site of a previously excised cutaneous neurofibroma in a patient without NF1. Given the rapidly progressive behavior and high mortality rates of MPNSTs, including superficial variants, timely recognition is critical, even in individuals without known genetic risk factors. These features underscore the need for prompt assessment of recurrent neurofibromas with evolving symptoms, and further research into MPNST pathogenesis in non-NF1 populations.
